# Prognostic role of urokinase plasminogen activator in hepatocellular carcinoma: A protocol for systematic review and meta analysis

**DOI:** 10.1097/MD.0000000000023841

**Published:** 2020-12-24

**Authors:** Pengxian Tao, Lei Gao, Haiyuan Li, Bofang Wang, Xuemei Li, Ying Zhang, Hao Chen

**Affiliations:** a The Department of Tumor Surgery, Lanzhou University Second Hospital; b The Second Clinical Medical College, Lanzhou University, Lanzhou; c Department of Laboratory Medicine, the First Medical Centre, Chinese PLA General Hospital, Beijing, China.

**Keywords:** systematic review, meta-analysis, urokinase plasminogen activator, hepatocellular carcinoma, protocol

## Abstract

**Background:**

Previous studies have showed that the high expression of urokinase plasminogen activator (uPA) in pathology and serology is closely related to the progression of hepatocellular carcinoma (HCC). However, there are no systematic reviews for these evidence, and the association between uPA and HCC is still not completely understood. Therefore, we will undertake a systematic review of the literature to summarize previous evidence regarding this topic, in order to clarify the prognostic significance of uPA in HCC.

**Methods and analysis:**

Studies comparing the HCC patients with high and low expression of uPA on the clinicopathological features and the prognosis are eligible for this review. Outcomes include all endpoints about survival and clinicopathological features. Prospective or retrospective primary studies which published in English will be included. Four databases of Medline, EMBASE, Web of Science, and the Cochrane Library will be systematically searched from their inception to Mar 2021 to retrieve relevant studies. Reference lists of included studies will be manually reviewed and grey literatures will be identified by Google Scholar. Two reviewers will independently screen the records and extract the information and data of the included studies. The Newcastle-Ottawa Scale will be used to assess the quality of included studies. Hazard ratio and 95% confidence interval will be pooled to assess the association between uPA expression and the prognosis. Pooled odds ratio and 95% confidence interval will be used for other outcomes. Heterogeneity will be assessed using the Cochrane *Q* test and *I*^2^ statistic, and a subgroup analysis will be performed if necessary. Grades of Recommendation, Assessment, Development and Evaluation method will be applied to assess the certainty of evidence.

**Ethics and dissemination:**

This protocol required information extracted from previously published articles. So, there is no ethical problem in this study. We plan to publish our findings in peer-reviewed journals and relevant conference proceedings.

**Systematic review registration:**

This study has been registered with the International Prospective Register of Systematic Reviews database (no.CRD42020150340).

## 1 Introduction

Hepatocellular carcinoma (HCC), the fifth and seventh most common cancer in adult man and female with more than 700,000 new cases and over 600,000 deaths worldwide per year.^[1,2]^ The distribution of HCC varies according to geographic location, high in Asian and sub-Saharan Africa, with incidence rates of over 20 per 100,000 individuals, and low in countries of northern Europe, Middle East, Oceania, North and South America.^[3]^

Earlier diagnosis is benefit for the prognostic for HCC. There are multiple methods for diagnosing, such as the alpha-fetoprotein serum values,^[4]^ alpha-fetoprotein-L3,^[4]^ DCP,^[5]^ circulating miRNAs,^[6]^ GP73, and GPC3.^[7]^ Moreover, numerous microRNAs and genes/proteins are involved in carcinogenesis and prognosis for HCC. Urokinase plasminogen activator (uPA) is 1 of this indexes, which is proved to relate with prognosis and earlier diagnosis of HCC.^[8]^ However, above mentioned indexes performed various specificity and sensitivity for HCC prognosis and recurrence. Thus, identifying the biological indicators of invasion and metastasis, especially with regard to the prognosis of HCC, is particularly important.

uPA is a serine protease of 55 kDa molecular mass, mainly composed of a growth factor-like domain, a kringle domain and a serine protease domain.^[9]^ In the tumor progression, uPA binds to its cell surface receptor uPAR, which then catalyzes the further transformation of pro-metalloproteinases into metalloproteinases.^[10]^ These activated metalloproteinases then promote the degradation of the extracellular matrix,^[11]^ and contribute to tumor cell invasion and metastasis at secondary tumor sites. During previous research, some case have showed that the high expression of uPA in pathology and serology is closely related to the poor prognosis of HCC,^[12,13]^ although the majority of published reports are of small or medium size. Obviously, heterogeneity in study design, data collection, documentation and presentation limit the accessibility of these data. So far, there is no a reliable evaluation system on overall survival and survival rate between uPA expression and HCC. However, there are no systematic reviews for these evidence, and the association between uPA and HCC is still not completely understood. Therefore, we will undertake a systematic review of the literature to summarize previous evidence regarding this topic, in order to clarify the prognostic significance of uPA in HCC.

## 2 Objectives

To determine the relationship between uPA expression and clinicopathological features in HCC patients, and to analyze the prognostic value of uPA in HCC.

## 3 Methods and analysis

### 3.1 Registration

This protocol was registered with International Prospective Register of Systematic Reviews database (CRD42020150340)

### 3.2 Participants

Patients with HCC will be considered for this review. The diagnosis is histologically confirmed. The patients received success hepatectomies, and tissue samples were preserved for the test of uPA expression. The stage and histological degree of tumours are determined according to the Barcelona Clinic Liver Cancer staging system^[14]^ and Edmondson's grade,^[15]^ respectively.

### 3.3 Exposure

The expression level of uPA in tissue is the exposure variable of this review. We will include studies detecting uPA expression in HCC tissues by the immunohistochemistry analysis. The expression level of uPA is defined as high and low expression based on the immunohistochemical staining. Studies should provide data comparing the HCC patient with high and low expression of uPA on the clinicopathological features and the prognosis.

### 3.4 Outcomes

Outcomes that indicate the associations of uPA expression with prognosis or clinicopathological features will be included for this review. Any outcomes about prognosis are eligible as long as sufficient information are provided to estimate the hazard ratio (HR). The clinicopathological features will include tumor differentiation, lymph node metastasis, tumor size, and TNM stage, and so on.

### 3.5 Studies

Prospective or retrospective published studies which evaluate the association between the uPA expression and HCC are eligible for this review. Studies will be excluded if they are letters, case reports, reviews, and animal studies. Non-English studies will also be excluded.

### 3.6 Information sources

Medline, EMBASE, Web of Science, and the Cochrane Library will be systematically searched from their inception to Mar 2021.

### 3.7 Search strategy

A combination of subject terms with free-text terms are used during the searches of 4 databases. The following search words is adopted for each database: (((tumour∗ or carcinom∗ or tumor∗ or malign∗ or neoplasm∗ or cancer∗) and (hepat∗ or liver)) or HCC) and (uPA or Urokinase∗ plasminogen or Urokinase-Type Plasminogen Activator). Other relevant articles which are not recorded in databases will be also manually reviewed from the reference lists of eligible studies. In addition, we will also search in Google Scholar to identify any grey literatures.

## 4 Study records

### 4.1 Data management

All study records are processed through EndNote X9, which can identify and remove duplicates. All extracted data are stored in a Microsoft Excel spreadsheet.

### 4.2 Study selection and data collection process

Two reviewers will independently assess the titles and abstracts of records identified by the literature searches. The full-text articles of any potentially relevant studies will be obtained. The same reviewers then will assess the full text of these potentially relevant studies for inclusion based on our inclusion criteria. Any disagreements will be solved by face-to-face discussion, with arbitration by a third researcher, when necessary. The data and information of the finally included study will be independently extracted by 2 reviewers using pre-piloted electronic data extraction forms. When differences in extracted data arise, 2 reviewers will discuss to reach consensus, or a third researched will be consulted if necessary.

### 4.3 Data items

The following data are extracted: author name, publication year, study location, study design, number of patients, follow-up period, outcome measures, baseline information of patients, clinicopathology (such as tumour differentiation, lymph node metastasis, tumour size, and TNM stage), HR, and 95% confidence interval (CI) related to uPA expression. Any items associated with a risk of bias will be also summarized.

### 4.4 Risk of bias in individual studies

The Newcastle-Ottawa Scale (NOS)^[16]^ will be applied to assess the quality of each included study. Scores of the NOS are split into 3 aspects: object selection, inter-group comparability, and outcome measurement. The NOS scores range from 0 to 9, and it is generally considered that an article with a score > 6 is of high quality.

### 4.5 Data synthesis

All statistical tests will be performed using the software STATA. HR and 95% confidence interval (CI) will be calculated for each study to assess the association between uPA expression and the prognosis. The HR and 95% CI of each study will be first directly extracted from the original full text. For studies where HR and 95% CI are not provided, data will be obtained from the survival curves using Engauge Digitizer, and the method from Tierney et al^[17]^ will be used to calculate the HR and 95% CI. The HR and 95% CI of individual study will be finally combined using the STATA software. Pooled HR > 1 indicates poor prognosis for the group of high uPA expression. The level of statistical significance is set at the 5%. The association between uPA expression and clinicopathological features is assessed using the pooled odds ratio and 95% CI. We will conduct qualitative statistics if a meta-analysis is not appropriate due to small number of studies or concerns regarding substantial variability. Statistical heterogeneity will be assessed using the Cochrane *Q* test and *I*^2^ statistic. A fixed effect model will be applied when the effects are assumed to be homogenous (*P* > .05, *I*^2^ ≤ 50%); otherwise, the random effect model will be adopted (*P* < .05, *I*^2^ ≥ 50%). To explore the source of heterogeneity, subgroup analysis will be performed through the HR obtain method, sample size, country, and study design. Sensitivity analysis will also be conducted to assess the stability of the results.

### 4.6 Meta-biases

If there were 10 or more studies included in each meta-analysis, we will assess the publication bias graphically by a funnel plot and statistically by the Egger test.^[18]^*P* < .05 is considered to indicate publication bias.

### 4.7 Confidence in cumulative evidence

We will assess the certainty of evidence obtained from this systematic review using the Grades of Recommendation, Assessment, Development, and Evaluation method.^[19]^

## 5 Discussion

uPA has inestimable potential for HCC prognostic marker. In the previous study, numerous of literatures have demonstrated that fibrinolytic system or uPA were positive or negative relation with prognosis for various cancer types.^[20–22]^ For instance, uPA/ PAI-1 as a molecular biomarker was recommend by the American Society of Clinical Oncology and the Arbeitsgemeinschaft Gynäkologische Onkologie (German Gynecological Oncology Group) guidelines to avoid unnecessary CT/X for moderate risk patients with primary breast cancer.^[23]^ At present, several studies have reported the association between uPA and HCC.^[24,25]^ However, small sample of individual study and uncertain quality of evidence limited the clinical application of these results.

Therefore, there is an urgent requirement to make a systematic review of studies on the prognostic significance of uPA for HCC. This article is a protocol of our systematic review, which presented the detailed description of review implement. The results of our review will be reported strictly following the PRISMA criteria. By integrating the data from previous articles, this review will objectively reveal the relationship between the expression of uPA and clinicopathological features of patients with HCC, and clarify the prognostic value of uPA for HCC.

The results of this upcoming study will help us understand the role of uPA for HCC, and it will provide a reference for developing a new strategy of prognosis assessment in HCC patients. It will also indicate the areas, where further research is necessary, before clinical application of uPA as a prognostic marker can be considered.

### 5.1 Reporting of the review

**Figure 1:**
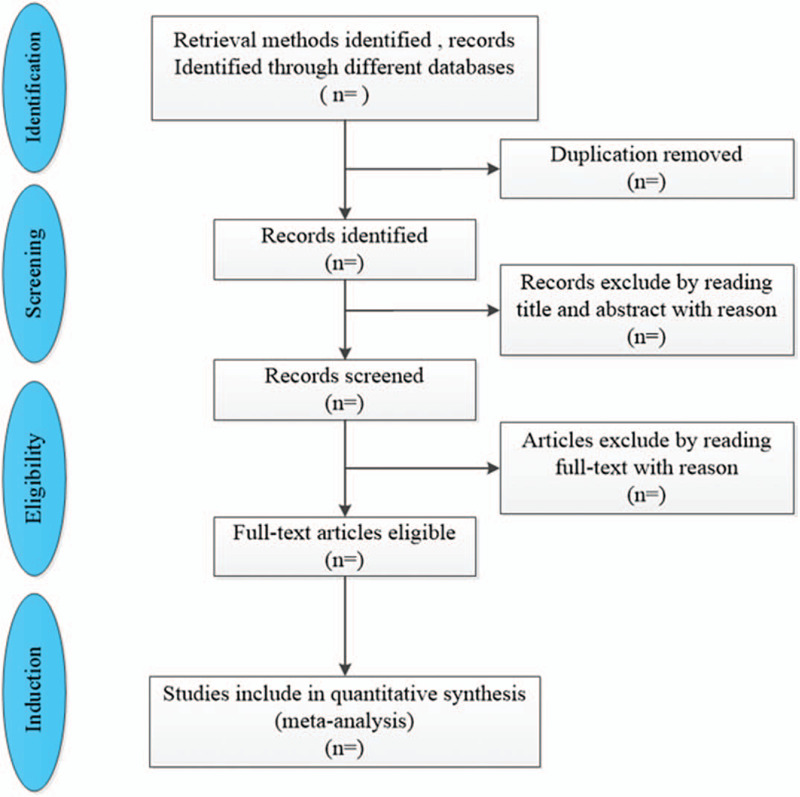
Flowchart: selection of studies for inclusion in meta-analysis.

The findings will be published as per PRISMA guidelines. A flow chart will be employed to outline the search producer (Fig. 1). Text description will be used to review the qualitative data of the included studies. Outputs of meta-analyses will be depicted in a forest plot and survival curve. Publication bias will be represented in the inverted funnel plot. The search strategy and quality appraisal tool will be provided in the supplement.

## Author contributions

PXT, YZ, and HC conceived the idea and plan for the study. PXT and LG wrote the manuscript of this protocol. LG, YZ and HYL designed the search strategy and planned the data extraction. BFW and XML made contributions in conceiving this research project. All authors revised and approved the final version of the manuscript.

**Conceptualization:** Pengxian Tao, Bofang Wang, Xuemei Li, Ying Zhang, Hao Chen.

**Methodology:** Lei Gao, Haiyuan Li, Ying Zhang, Hao Chen.

**Writing – original draft:** Pengxian Tao, Lei Gao.

**Writing – review & editing:** Haiyuan Li, Bofang Wang, Xuemei Li, Ying Zhang, Hao Chen.

## References

[1] Biselli M Garuti F Neri A Hepatocellular carcinoma surveillance: an open question. Hepatobiliary Surg Nutr 2019;8:431–2. 31489324 10.21037/hbsn.2019.02.10 PMC6700004

[2] Sagnelli E Macera M Russo A Epidemiological and etiological variations in hepatocellular carcinoma. Infection 2019;7–17. 10.1007/s15010-019-01345-y 31347138

[3] Cheung TT Management of hepatocellular carcinoma: from bench to bedside and beyond. Transl Gastroenterol Hepatol 2019;4:54. 31463413 10.21037/tgh.2019.07.01 PMC6691057

[4] Alqahtani A Khan Z Alloghbi A Hepatocellular carcinoma: molecular mechanisms and targeted therapies. Medicina 2019;55:10.3390/medicina55090526 PMC6780754 31450841

[5] Sumi A Akiba J Ogasawara S Des-gamma-carboxyprothrombin (DCP) and NX-DCP expressions and their relationship with clinicopathological features in hepatocellular carcinoma. PloS One 2015;10:e0118452. 25739032 10.1371/journal.pone.0118452 PMC4349810

[6] Wang S Wu J Shen S Evaluation of different blood circulating mirnas for hepatocellular carcinoma diagnosis. J Nanosci Nanotechnol 2020;20:1983–8. 31492371 10.1166/jnn.2020.17160

[7] Yao S Zhang J Chen H Diagnostic value of immunohistochemical staining of GP73, GPC3, DCP, CD34, CD31, and reticulin staining in hepatocellular carcinoma. J Histochem Cytochem 2013;61:639–48. 23686365 10.1369/0022155413492771 PMC3753886

[8] Madunic J The urokinase plasminogen activator system in human cancers: an overview of its prognostic and predictive role. Thromb Haemost 2018;118:2020–36. 30419600 10.1055/s-0038-1675399

[9] Ahmad Akhoundi MS Rokn A Bagheri R Urokinase-plasminogen activator protects periodontal ligament fibroblast from oxidative induced-apoptosis and DNA damage. J Periodontal Res 2018;53:861–9. 29920670 10.1111/jre.12576

[10] Yeh CB Yu YL Lin CW Terminalia catappa attenuates urokinasetype plasminogen activator expression through Erk pathways in Hepatocellular carcinoma. BMC Complement Altern Med 2014;14:141. 24886639 10.1186/1472-6882-14-141 PMC4012530

[11] Panich T Tragoolpua K Pata S Downregulation of extracellular matrix metalloproteinase inducer by scFv-M6-1B9 intrabody suppresses cervical cancer invasion through inhibition of urokinase-type plasminogen activator. Cancer Biother Radiopharm 2017;32:1–8. 28118037 10.1089/cbr.2016.2126

[12] Itoh T Hayashi Y Kanamaru T Clinical significance of urokinase-type plasminogen activator activity in hepatocellular carcinoma. J Gastroenterol Hepatol 2000;15:422–30. 10824888 10.1046/j.1440-1746.2000.02150.x

[13] Morita Y Hayashi Y Wang Y Expression of urokinase-type plasminogen activator receptor in hepatocellular carcinoma. Hepatology 1997;25:856–61. 9096588 10.1002/hep.510250412

[14] Llovet JM Bru C Bruix J Prognosis of hepatocellular carcinoma: the BCLC staging classification. Semin Liver Dis 1999;19:329–38. 10518312 10.1055/s-2007-1007122

[15] Edmondson HA Steiner PE Primary carcinoma of the liver: a study of 100 cases among 48,900 necropsies. Cancer 1954;7:462–503. 13160935 10.1002/1097-0142(195405)7:3<462::aid-cncr2820070308>3.0.co;2-e

[16] Stang A Critical evaluation of the Newcastle-Ottawa scale for the assessment of the quality of nonrandomized studies in meta-analyses. Eur J Epidemiol 2010;25:603–5. 20652370 10.1007/s10654-010-9491-z

[17] Tierney JF Stewart LA Ghersi D Practical methods for incorporating summary time-to-event data into meta-analysis. Trials 2007;8:16. 17555582 10.1186/1745-6215-8-16 PMC1920534

[18] Duval S Tweedie R Trim and fill: A simple funnel-plot-based method of testing and adjusting for publication bias in meta-analysis. Biometrics 2000;56:455–63. 10877304 10.1111/j.0006-341x.2000.00455.x

[19] Iorio A Spencer FA Falavigna M Use of GRADE for assessment of evidence about prognosis: rating confidence in estimates of event rates in broad categories of patients. BMJ 2015;350:h870. 25775931 10.1136/bmj.h870

[20] Mahmood N Mihalcioiu C Rabbani SA Multifaceted role of the urokinase-type plasminogen activator (uPA) and its receptor (uPAR): diagnostic, prognostic, and therapeutic applications. Front Oncol 2018;8:24. 29484286 10.3389/fonc.2018.00024 PMC5816037

[21] Song KS Lee A Choi JR Diagnostic efficacy of plasma urokinasetype plasminogen activator and plasminogen activator inhibitor-2 in differentiation of hepatocellular carcinoma from cirrhosis. Thromb Haemost 1995;74:864–7. 8571313

[22] Zheng Q Tang ZY Xue Q Invasion and metastasis of hepatocellular carcinoma in relation to urokinase-type plasminogen activator, its receptor and inhibitor. J Cancer Res Clin Oncol 2000;126:641–6. 11079728 10.1007/s004320000146 PMC12165145

[23] Jacobs VR Augustin D Wischnik A Concordance rates of biomarkers uPA and PAI-1 results in primary breast cancer vs. consecutive tumor board decision and therapy performed in clinical hospital routine: Results of a prospective multi-center study at certified breast centers. Breast 2016;29:208–12. 27344290 10.1016/j.breast.2016.06.014

[24] Dubuisson L Monvoisin A Nielsen BS Expression and cellular localization of the urokinase-type plasminogen activator and its receptor in human hepatocellular carcinoma. J Pathol 2000;190:190–5. 10657018 10.1002/(SICI)1096-9896(200002)190:2<190::AID-PATH511>3.0.CO;2-H

[25] Sato S Higashi T Ouguchi S Elevated urokinase-type plasminogen activator plasma levels are associated with deterioration of liver function but not with hepatocellular carcinoma. J Gastroenterol 1994;29:745–50. 7874270 10.1007/BF02349281

[26] Shamseer L Moher D Clarke M Preferred reporting items for systematic review and meta-analysis protocols (PRISMA-P) 2015: elaboration and explanation. BMJ 2015;350:g7647. 25555855 10.1136/bmj.g7647

